# Disseminated *Mycobacterium tilburgii* infection in a person with AIDS: A case report

**DOI:** 10.1016/j.heliyon.2024.e35616

**Published:** 2024-08-02

**Authors:** Man Yuan, Guowei Dong, Ning Han, Libo Yan, Hong Tang

**Affiliations:** a Center of Infectious Diseases, West China Hospital of Sichuan University, Chengdu, China; b Department of Infectious Diseases, Chengdu Seventh People's Hospital (Affiliated Cancer Hospital of Chengdu Medical College), Chengdu, China

**Keywords:** *Mycobacterium tilburgii*, Acquired immune deficiency syndrome, Metagenomics next-generation sequencing, Case report

## Abstract

**Background:**

*Mycobacterium tilburgii* is a nonculturable, nontuberculous mycobacterium that occasionally causes serious infections in individuals with immune deficiencies. Owing to its nonculturable nature, its antimicrobial susceptibility has not been assessed, and the optimal treatment regimen is unclear. Herein, we report a case of disseminated *M. tilburgii* infection in a person with AIDS, identified using metagenomics next-generation sequencing (mNGS) and polymerase chain reaction (PCR).

**Case presentation:**

A 33-year-old man presented with a 3-month history of abdominal pain, lymphadenopathy, intermittent night hot flashes, night sweats, and weight loss. No pathogen was detected during initial routine investigations. *M. tilburgii* was subsequently identified in a left cervical lymph node sample using mNGS. Furthermore, *M. tilburgii* infection was detected in a bone marrow sample based on PCR of *16S rRNA* and *hsp65* gene sequencing. The person was treated with a combination of moxifloxacin, clarithromycin, ethambutol, rifabutin, and amikacin. The laboratory results improved, and the patient's symptoms resolved.

**Conclusion:**

*M. tilburgii* may be missed in diagnostic tests because it cannot be grown using routine culture techniques. Early diagnosis and timely and effective treatment are critical in patients with *M. tilburgii* infection; therefore, molecular techniques are recommended for patients with suspected *M. tilburgii* infection.

## Highlights

• *M. tilburgii* is a nonculturable non-tuberculous mycobacterial.
• The mNGS technique can detect pathogens that are difficult to cultivate.
• We report a case with disseminated *M. tilburgii* infection proved by mNGS.

## 1 Introduction

*Mycobacterium tilburgii* was first identified in an immunocompetent adult patient from Tilburg in the Netherlands in 1995 [1]. It is related to *Mycobacterium simiae* complex which causes infections in the lungs, lymph nodes, gastrointestinal tract, and bone marrow [2,3]. It has never been isolated on culture and its detection relies on direct sequencing. Herein, we report a case of disseminated *M. tilburgii* infection in a person with AIDS, confirmed by metagenomic next-generation sequencing (mNGS) and polymerase chain reaction (PCR).

## 2 Case presentation

In July 2023, A 33-year-old man was admitted to our hospital with a 3-month history of abdominal pain, lymphadenopathy, intermittent hot flushes (not exceeding 38.5 °C), night sweats, and a weight loss of 15 kg. He had tested positive for human immunodeficiency virus (HIV) in 2008 and had commenced antiretroviral therapy (ART) with tenofovir/lamivudine/efavirenz in 2018. In August 2022, his CD4 count dropped to 200/μL. He had a good drug compliance and his local doctors considered the possibility of drug resistance and changed the ART regimen to lopinavir/ritonavir/tenofovir/lamivudine in March 2023. His hemoglobin (Hb) level decreased from 110 g/L at the beginning of April 2023, to 77 g/L 6 weeks later. His doctors considered the anemia to be related to the ART regimen and changed it to bictegravir/emtricitabine/tenofovir alafenamide.

Abdominal computed tomography (CT) performed in March 2023 showed multiple enlarged lymph nodes up to 3 cm in diameter at the root of the mesentery and adjacent to the abdominal aorta. In May 2023, a needle biopsy of an abdominal lymph node showed inflammatory granulomatous tissue and infiltration of macrophage-like cells. Furthermore, excisional biopsy of a left cervical lymph node revealed chronic suppurative inflammation. However, the findings were nonspecific and did not provide sufficient evidence to reach a diagnosis.

The patient was admitted to our hospital in July 2023 for further investigation. On physical examination, his body temperature was 36.5 °C, with a pulse of 100 beats per minute, blood pressure of 95/62 mmHg, and lymphadenopathy of the neck, armpits, and groin. He had recently stopped smoking after a cumulative cigarette consumption of 10 pack-years. The liver enzymes and lactate dehydrogenase levels were normal. Hematology showed an Hb level of 66 g/L, and a white blood cells count of 3.84 × 10^9^/L, 73 % neutrophils, and 5 % lymphocytes. His serum albumin level was 30.0 g/L. His HIV viral load was 2.15E+2 copies/mL and his CD4 count was 29 cells/μL. He had an elevated erythrocyte sedimentation rate of 94 mm/h, and laboratory tests showed elevated levels of procalcitonin (0.35 ng/mL), and C-reactive protein (67.1 mg/L). Cytomegalovirus and Epstein-Barr virus DNA levels were both <50 copies/mL. All other screening tests to identify a cause of infection, including hepatitis B, and C, syphilis, and interferon-γ release assays, serum (1,3)-beta-D-glucan, serum galactomannan, sputum PCR testing for *Pneumocystis jirovecii* DNA, serum

*cryptococcal* antigen, and blood culture were all negative.

**Fig. 1:**
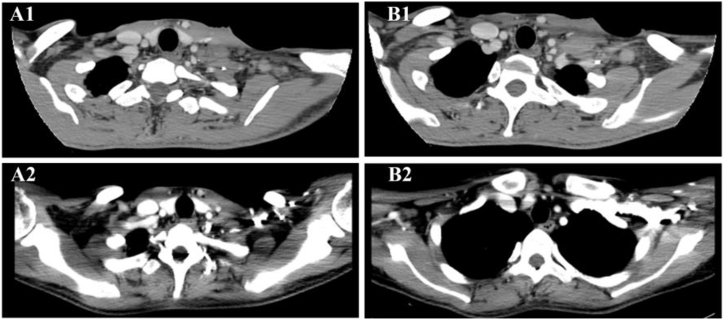
Comparison of image findings pre-and post-treatment. (a1, b1) The neck CT on July 2023, indicated enlarged lymph nodes in the neck. (a2, b2) After antimycobacterial treatment, the neck CT on November 2023, showed some of the lymph nodes shrank.

**Fig. 2:**
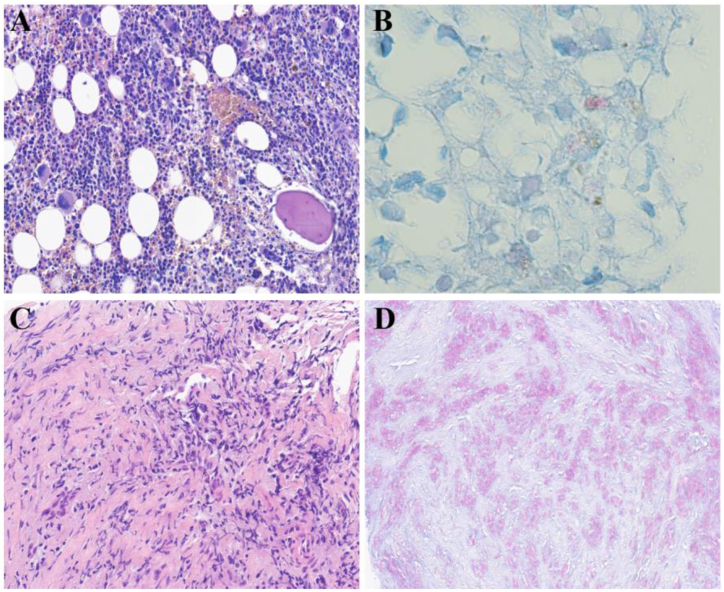
Histopathology images. (a, b) A bone marrow: Hematoxylin and eosin stain and Ziehl-Neelsen stain. (c, d) A left cervical lymph node: Hematoxylin and eosin stain and Ziehl-Neelsen stain.

Lymph node ultrasonography showed lymphadenopathy of the neck (largest lymph node approximately 39 × 15 × 35 mm), supraclavicular region (largest lymph node: 42 × 22 × 33 mm), armpit (largest lymph node: 17 × 6 mm), and groin (largest lymph node: 18 × 3 mm). CT revealed multiple enlarged lymph nodes in the neck, and mesenteric, and para-aortic regions (Fig. 1). Lymphoma was suspected; therefore, we performed a bone marrow biopsy, which showed acid-fast (AF) bacilli, *Mycobacterium tuberculosis* (MTB)-DNA negativity, and an absence of proliferating cells (Fig. 2). In addition, the peripheral blood showed a lack of proliferating cells. The local hospital sent the excised left cervical lymph node specimen to our hospital for further histological examination. It showed Lymph node necrosis, abscess formation, inflammatory granulomatous tissue, AF bacilli, and was confirmed to be MTB-DNA negative. These findings led us to suspect nontuberculous mycobacterial (NTM) infection. Next, we performed a left cervical lymph node puncture biopsy, which showed histiocyte proliferative lesions, AF bacilli, and EBER1/2 negativity, and the cultures remained negative (Fig. 2). A lymph node specimen was sent to Vision Medical Laboratory (Chengdu, China) for further testing. Within 24 hours of the laboratory receiving the specimen, mNGS showed 742,828 specific sequences that were 99 % identical to the genomic sequence of *M. tilburgii*. (SRA accession number: PRJNA1109638; Supplementary). The initial bone marrow specimen was sent to our hospital's center for pathogen microbiology the following day for further testing. PCR of *16S rRNA* and *hsp65* gene sequencing of the initial bone marrow specimen confirmed that the bone marrow was positive for *M. tilburgii* infection within 24 hours of the laboratory receiving the specimen (Supplementary).

**Fig. 3:**
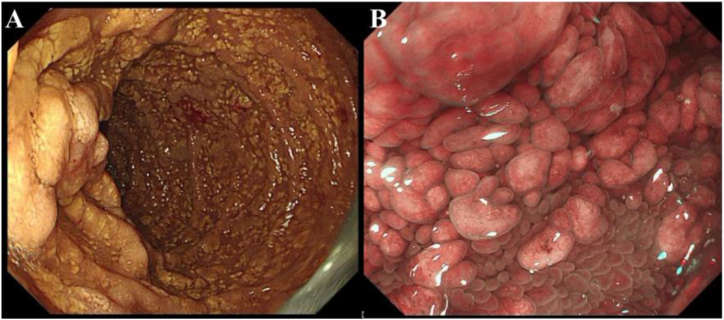
Esophagogastroduodenoscopy result. (a) Esophagogastroduodenoscopy revealed widespread yellow plaques in the descending duodenum. (b) The magnifying endoscopy with narrow-band imaging observed the yellow plaques with villous changes.

Based on these findings, the person was diagnosed with disseminated *M. tilburgii* infection. Because *M. tilburgii* cannot be cultured, no information is available on drug susceptibility or the optimal treatment option. Based on previous case reports, we initiated empirical antimycobacterial treatment with moxifloxacin 400 mg daily, clarithromycin 500 mg twice daily, ethambutol 750 mg daily, and rifabutin 300 mg daily, and changed the ART regimen to lamivudine/dolutegravir/tenofovir at the same time to prevent drug-drug interactions. One week later, the patient's abdominal pain, intermittent fever, and night sweats had resolved. One month later, his Hb level had increased to 117 g/L and he experienced no side effects of treatment. Four months after starting treatment (November 2023), he had gained of 10.5 kg in body weight. His HIV viral load was 4.22E+1 copies/mL and CD4 count was 36 cells/μL. PCR testing of a bone marrow specimen for *16S rRNA* and *hsp65* was negative, suggesting an absence of *M. tilburgii*. However, the patients continued to experience intermittent abdominal pain. Neck and abdominal CT revealed shrinkage of some of the lymph nodes but newly developed intestinal wall thickening (Fig. 1). Esophagogastroduodenoscopy revealed widespread yellow plaques in the descending duodenum with villous changes (Fig. 3). A duodenal biopsy specimen showed an accumulation of foam cells in the submucosal layer, AF bacilli, and MTB-DNA negativity. Therefore, we diagnosed duodenal *M. tilburgii* infection and added amikacin to the treatment. After 2 months of amikacin therapy, the intermittent abdominal pain resolved, with no side effects of amikacin such as hearing loss. Hematology showed an Hb level of 115 g/L. Patient follow-up is ongoing.

## 3 Discussion

Only 15 cases of *M. tilburgii* infection have been reported to date, most of which involved immunocompromised hosts (such as people living with HIV, people on chronic corticosteroid therapy, and people with interleukin-12 or interferon-gamma receptor 1 deficiencies) [3]. Five cases have been reported in people living with HIV [[4], [5], [6], [7], [8]]. Of these, four had duodenal involvement, two had lymph node involvement, one had pulmonary nodules, and one had bone marrow involvement. All cases reported in people living with HIV were associated with low CD4 counts, ranging from 6 to 37 cells/μL. Ethambutol, clarithromycin, and rifabutin are the most widely used antibiotics for treatment of *M. tilburgii* infection. All patients recovered, except for one patient who died of malignant lymphoma in the brain [7]. A published case from Germany described infection of the bone marrow [8]. The patient underwent 4 months of NTM therapy and subsequently developed anemia (Hb 8.2 g/dL). A bone marrow biopsy confirmed *M. tilburgii* infection. In contrast, our patient had anemia in the early stage of the disease before initiating anti-NTM therapy. These findings suggest that bone marrow involvement should be suspected in patients with *M. tilburgii* infection and anemia.

As NTM are intracellular parasitic microorganisms, their detection is complicated. *M. tilburgii* may be missed using standard diagnostic tests, because it cannot be cultured by routine techniques. Moreover, antimicrobial susceptibility has not yet been assessed, and the optimal treatment regimen is unclear. To our knowledge, this is the first report of the use of mNGS to assist with the diagnosis of *M. tilburgii* infection in a person with AIDS. Previous studies have relied primarily on *16S rRNA* sequencing. The mNGS technique is increasingly used to detect pathogens that are difficult to culture or cannot be cultured [9]. It enables rapid detection and comprehensive identification of bacteria, fungi, viruses, and parasites, and is especially useful for identifying mycobacteria, anaerobes, atypical pathogens, and viruses [9]. It is recommended in situations involving patients with difficult or complicated infectious diseases, including those who are acutely and critically ill, suffering from immunodeficiency, as well as outbreaks of unknown origin [9]. Compared with targeted tests, such as PCR, mNGS does not target any specific pathogen. However, mNGS is unlikely to replace PCR because of its limitations, such as high cost (US $456 for DNA detection and $801 for both DNA and RNA) and lack of a standard testing protocol.

## 4 Conclusion

This report describes a case of disseminated *M. tilburgii* infection in a Chinese man with AIDS that was confirmed using mNGS and PCR. Early diagnosis and timely and effective treatment are critical in treating patients with *M. tilburgii* infection; therefore, molecular techniques such as mNGS are recommended for diagnosis in patients with suspected *M. tilburgii* infection. However, the utility of mNGS extends beyond *M. tilburgii* to potentially include other NTM strains that are challenging or impossible to culture. The patient was successfully treated with prolonged treatment with a combination of moxifloxacin, clarithromycin, ethambutol, rifabutin, and amikacin. However, long-term follow-up is required to assess whether the patient has been cured. Further studies are required to determine the most effective treatment.

## Ethics statement

This study was approved by the Ethics Committee of the West China Hospital of Sichuan University (approval number: 2020–450). Informed consent was obtained from the patient for the publication of all images, clinical data and other data included in the manuscript. This study complied with relevant guidelines and regulations.

## Data availability statement

Data included in article/supp. material/referenced in article. And the mNGS sequencing data supporting the conclusion of this article is available in the [NCBI SRA] database, [Accession number: PRJNA1109638]. Further inquiries can be directed to the corresponding authors on reasonable request.

## Funding

This work was supported by the Science and Technology project of the 10.13039/501100020207Health Commission of Sichuan Province (grant no. 23009).

## CRediT authorship contribution statement

**Man Yuan:** Writing – original draft. **Guowei Dong:** Data curation. **Ning Han:** Data curation. **Libo Yan:** Writing – review & editing. **Hong Tang:** Writing – review & editing.

## Declaration of competing interest

The authors declare that they have no known competing financial interests or personal relationships that could have appeared to influence the work reported in this paper.
