# Supplementary material for: Disseminated Mycobacterium tilburgii infection in a person with AIDS: A case report
Source: Heliyon. 2024 Aug 2;10(15):e35616. doi: 10.1016/j.heliyon.2024.e35616 (PMC11336850; doi:10.1016/j.heliyon.2024.e35616)

**Disseminated *Mycobacterium tilburgii* infection in a person with AIDS: A case report**

**Supplementary**

**Supplementary Methods**

**Metagenomic Next-Generation Sequencing (mNGS)**

DNA extraction, Library construction, Capture hybirdtion and Sequencing

A standard operating procedure of the DNA-based mNGS method was developed for the diagnosis of pathogen. Briefly, 1 mL of sample was centrifuged at 12,000 × g for 5 min to collect the pathogens and human cells. Next, 50 µL of precipitate underwent depletion of host nucleic acid using 1 U Benzonase (Sigma) and 0.5% Tween 20 (Sigma) and incubated at 37°C for 5 min. Terminal buffer (400 µL) was added to stop the reaction. A total of 600 µl of the mixture was transferred to new tubes containing 500 µL of ceramic beads for bead beating using a Minilys Personal TGrinder H24 Homogenizer (catalog number: OSE-TH-01, Tiangen, China). Then, the nucleic acid from 400 µL of pretreated samples was extracted and eluted in 60 µL elution buffer using a QIAamp UCP Pathogen Mini Kit (catalog number: 50214, Qiagen, Germany). The extracted DNA was quantified using a Qubit dsDNA HS Assay Kit (catalog number: Q32854, Invitrogen, USA) [1, 2].

10 µL of purified RNA was used for cDNA generation. DNA/cDNA were constructed library using the KAPA low throughput library construction kit (KAPA Biosystems, U.S.A.) according to the manufacturer’s instructions [2]. An aliquot of 750-ng library from each sample was used for hybrid capture-based enrichment of microbial probe one rounds of hybridization (SeqCap EZ Library, Roche, U.S.A). Probe designed were used CATCH pipeline with default parameters based on pathogen genome [3].

Bioinformatic analysis

Trimmomatic was used to remove low-quality reads, adapter contamination, duplicate reads, and those shorter than 70 bp [4]. Low-complexity reads were removed by Kcomplexity using default parameters. The human sequence data were identified and excluded by mapping to a human reference genome (hg38) using SNAP v1.0beta.18 [5]. To construct the microbial genome database, pathogens and their genomes or assemblies were selected following the Kraken 2 criteria for selecting representative assemblies for microorganisms (bacteria, viruses, fungi, protozoa, and other multicellular eukaryotic pathogens) from the NCBI Assembly and Genome databases (https://benlangmead.github.io/aws-indexes/k2) [6]. Microbial reads were aligned to the database using Burrows‒Wheeler Aligner software [7]. We defined that reads with 90% identity of reference were mapped reads. In addition, reads with multiple locus alignments within the same genus were excluded from the secondary analysis. Only reads mapped to the genome within the same species were considered.

We normalized the sequencing reads RPTM to eliminate the errors caused by various sequencing depths among samples [8]. For microorganisms without culture isolates, the RPTM mean value and standard deviation of this microorganism were calculated, and the RPTM (mean + 2SD) was set as a positive cutoff value [9].

The clinical reportable range (CRR) for pathogens was established according to the following three references indicated in a previous study [9]: Ⅰ. Johns Hopkins ABX Guide (https://www.hopkinsguides.com/hopkins/index/Johns_Hopkins_ABX_Guide/Pathogens), Ⅱ. Manual of Clinical Microbiology [10], and Ⅲ. clinical case reports or research articles published in peer-reviewed journals.

Quality Control

To monitor the sources of potential contamination, both NC and sterile deionized water, which served as non-template controls, were prepared in parallel with other samples in each batch [11]. In addition, we used sterile cotton swabs dipped in sterile deionized water to wipe the surfaces of the centrifuge and biosafety cabinet to generate the background microorganism list in our laboratory.

Results

Lymph node biopsy yielded a total of 742,828 specific sequences reads mapped to *M. tilburgii* in the reference database, and the coverage of *M. tilburgii* genome was 99.3054% (Supplementary Table 1 and Fig. 1).

**PCR Testing**

PCR using the *16S rRNA* gene primers followed by sequencing revealed a sequence of 467 bases. The obtained sequence was compared to those stored in GenBank using the Basic Local Alignment Search Tool (BLAST; NCBI, Bethesda, MD) and was shown to be 100% identical to the *16S rRNA* gene sequence of *M. tilburgii* (accession number AJ580826.1).

PCR using *hsp65* gene primers, and the obtained sequences (373 bases) was shown to be 100% identical to the *hsp65* gene sequence of *M. tilburgii* (accession number HM588695.1).

**REFERENCES**

1. Amar Y, Lagkouvardos I, Silva RL, Ishola OA, Foesel BU, Kublik S, et al. Pre-digest of unprotected DNA by Benzonase improves the representation of living skin bacteria and efficiently depletes host DNA, Microbiome. 9 (1) (2021) 123. doi:10.1186/s40168-021-01067-0
2. Zhou Z, Ren L, Zhang L, Zhong J, Xiao Y, Jia Z, et al. Heightened Innate Immune Responses in the Respiratory Tract of COVID-19 Patients, Cell Host Microbe. 27 (6) (2020) 883-890.e2. doi:10.1016/j.chom.2020.04.017
3. Metsky HC, Siddle KJ, Gladden-Young A, Qu J, Yang DK, Brehio P, et al. Capturing sequence diversity in metagenomes with comprehensive and scalable probe design, Nat Biotechnol. 37 (2) ( 2019) 160-168. doi:10.1038/s41587-018-0006-x
4. Bolger AM, Lohse M, Usadel B. Trimmomatic: a flexible trimmer for Illumina sequence data, Bioinformatics. 30 (15) (2014) 2114-2120. doi:10.1093/bioinformatics/btu170
5. Matei Zaharia WJB, Kristal Curtis, Armando Fox, David Patterson, Scott Shenker, Ion Stoica, et al. Faster and More Accurate Sequence Alignment with SNAP 2011. https://arxiv.org/abs/1111.5572.
6. Assembly Anomalies and Other Reasons a Genome Assembly may be Excluded from RefSeq: National Center for Biotechnology Information. https://www.ncbi.nlm.nih.gov/assembly/help/anomnotrefseq/.
7. Li H, Durbin R. Fast and accurate short read alignment with Burrows-Wheeler transform, Bioinformatics. 25 (14) (2009) 1754-1760. doi:10.1093/bioinformatics/btp324
8. Unal I. Defining an Optimal Cut-Point Value in ROC Analysis: An Alternative Approach, Comput Math Methods Med. 2017 (2017) 3762651. doi:10.1155/2017/3762651
9. Jing C, Chen H, Liang Y, Zhong Y, Wang Q, Li L, et al. Clinical Evaluation of an Improved Metagenomic Next-Generation Sequencing Test for the Diagnosis of Bloodstream Infections, Clin Chem. 67 (9) (2021) 1282-1283. doi:10.1093/clinchem/hvab061
10. Manual of Clinical Microbiology, 12th Edition. https://www.clinmicronow.org/doi/book/10.1128/9781683670438.MCM
11. Miller S, Naccache SN, Samayoa E, Messacar K, Arevalo S, Federman S, et al. Laboratory validation of a clinical metagenomic sequencing assay for pathogen detection in cerebrospinal fluid, Genome Res. 29 (5) (2019) 831-842. doi:10.1101/gr.238170.118

**Supplementary Table 1.** Potentially pathogenic microorganism(s) detected by metagenomic next-generation sequencing in a left cervical lymph node sample.

|  | Genus | | |  | Species | |
| --- | --- | --- | --- | --- | --- | --- |
| Type | Name | Relative abundance | Sequence no. |  | Name | Sequence no. |
| G+ | *Mycobacterium* | 95.3% | 1,123,132 |  | *Mycobacterium tilburgii* | 742,828 |

**Supplementary Figure 1.** Results of metagenomic next-generation sequencing of *Mycobacterium tilburgii* in a left cervical lymph node sample.


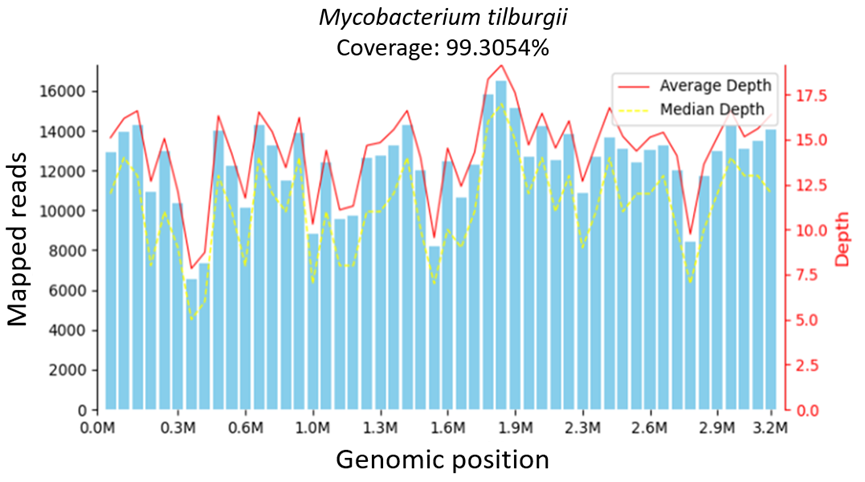

Supplement: Multimedia component 1 [file mmc1.docx]
